# Supplementary figures and images for: Dual sensory impairments in companion dogs: Prevalence and relationship to cognitive impairment
Source: PLoS One. 2024 Oct 16;19(10):e0310299. doi: 10.1371/journal.pone.0310299 (PMC11482676; doi:10.1371/journal.pone.0310299)

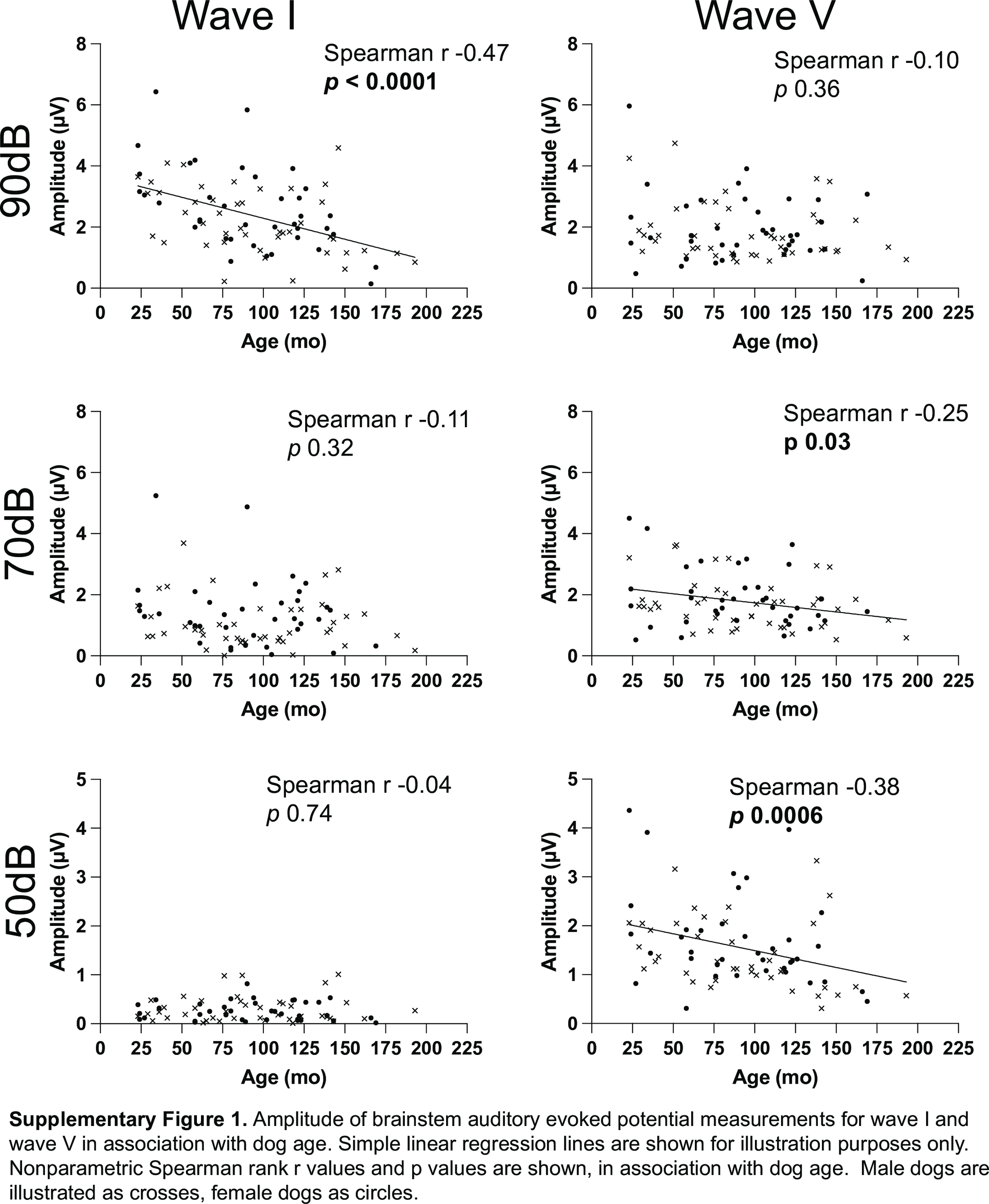

Supplement: S1 Fig — Amplitude of brainstem auditory evoked potential measurements for wave I and wave V in association with dog age. Simple linear regression lines are shown for illustration purposes only. Nonparametric Spearman rank r values and p values are shown, in association with dog age. Male dogs are illustrated as crosses, female dogs as circles. (TIF) [file pone.0310299.s001.tif]

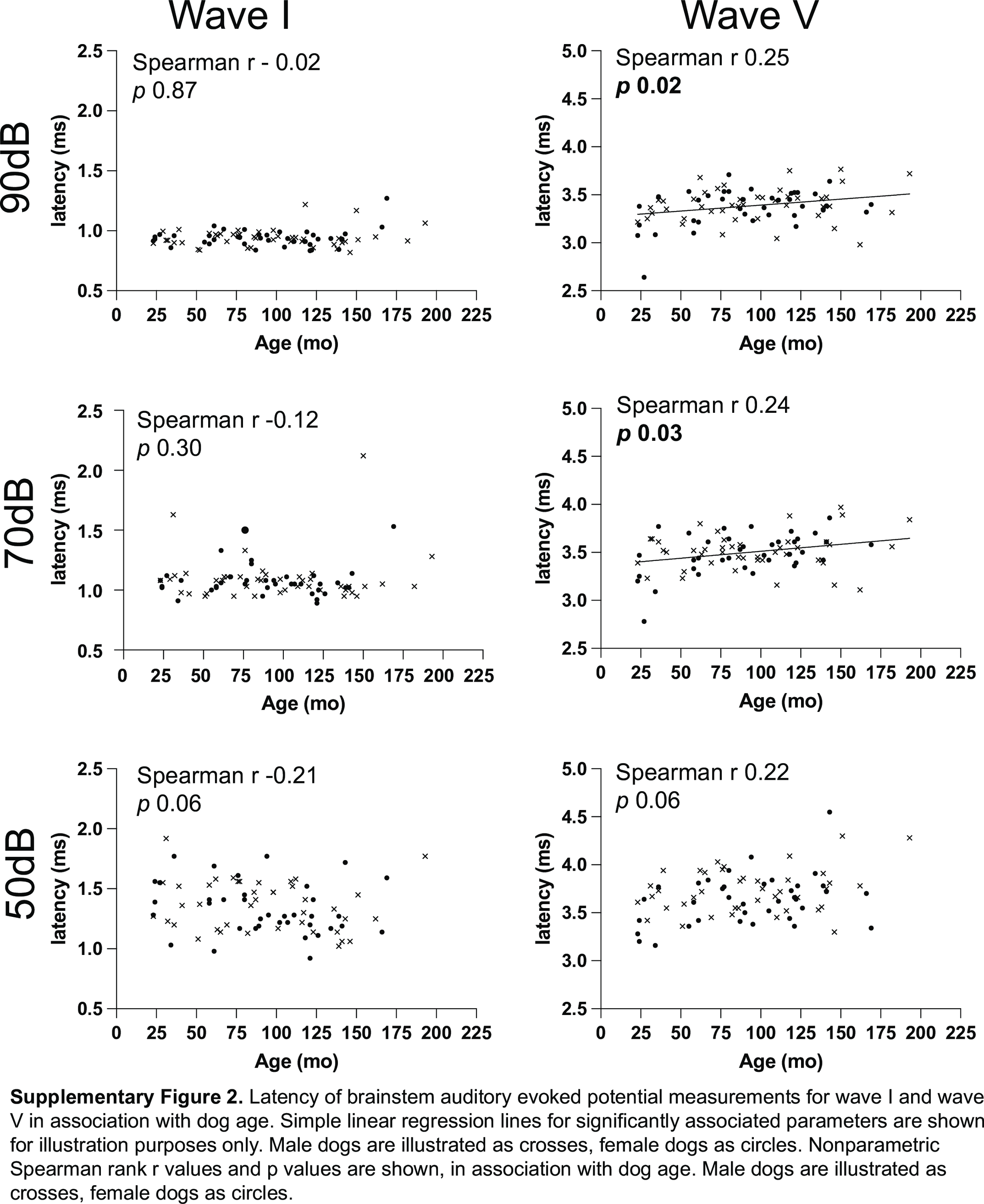

Supplement: S2 Fig — Latency of brainstem auditory evoked potential measurements for wave I and wave V in association with dog age. Simple linear regression lines for significantly associated parameters are shown for illustration purposes only. Male dogs are illustrated as crosses, female dogs as circles. Nonparametric Spearman rank r values and p values are shown, in association with dog age. Male dogs are illustrated as crosses, female dogs as circles. (TIF) [file pone.0310299.s002.tif]

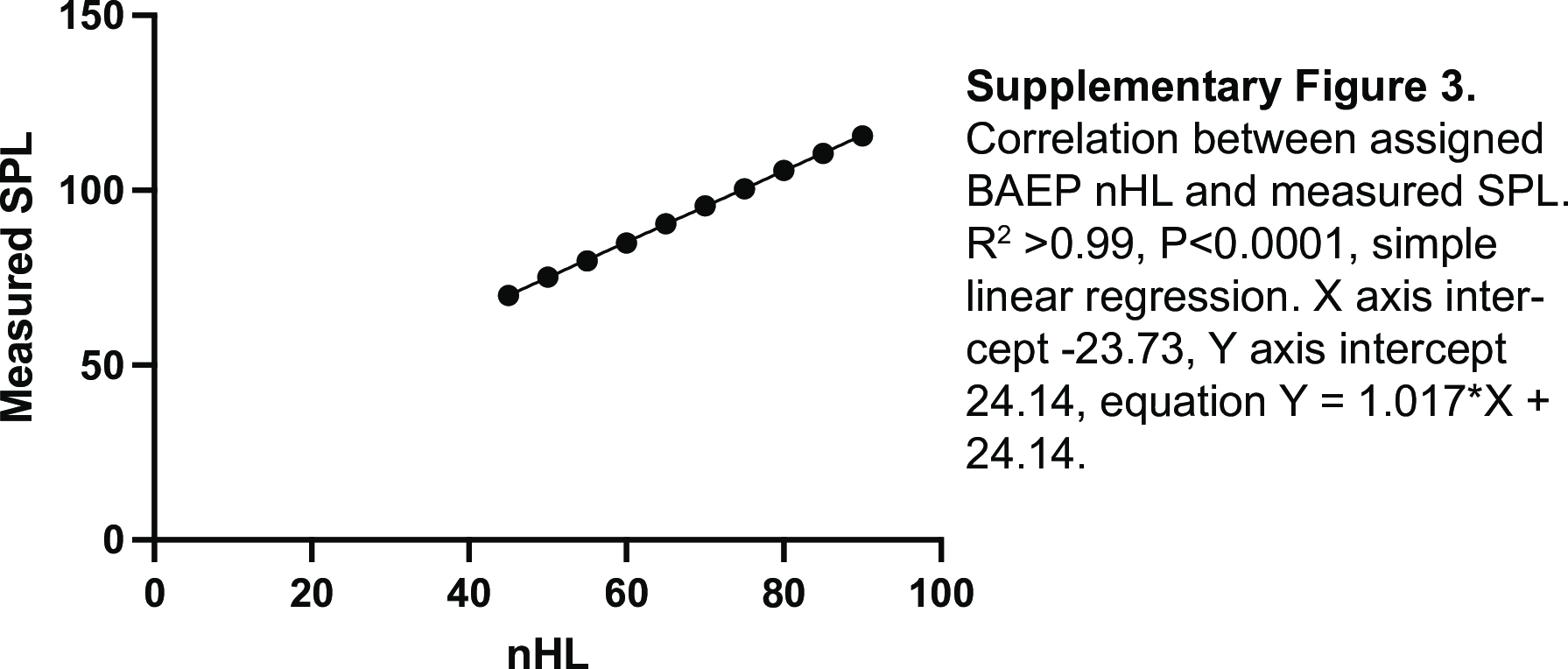

Supplement: S3 Fig — Correlation between assigned BAEP nHL and measured SPL. R2 >0.99, P<0.0001, simple linear regression. X axis intercept -23.73, Y axis intercept 24.14, equation Y = 1.017*X + 24.14. (TIF) [file pone.0310299.s003.tif]

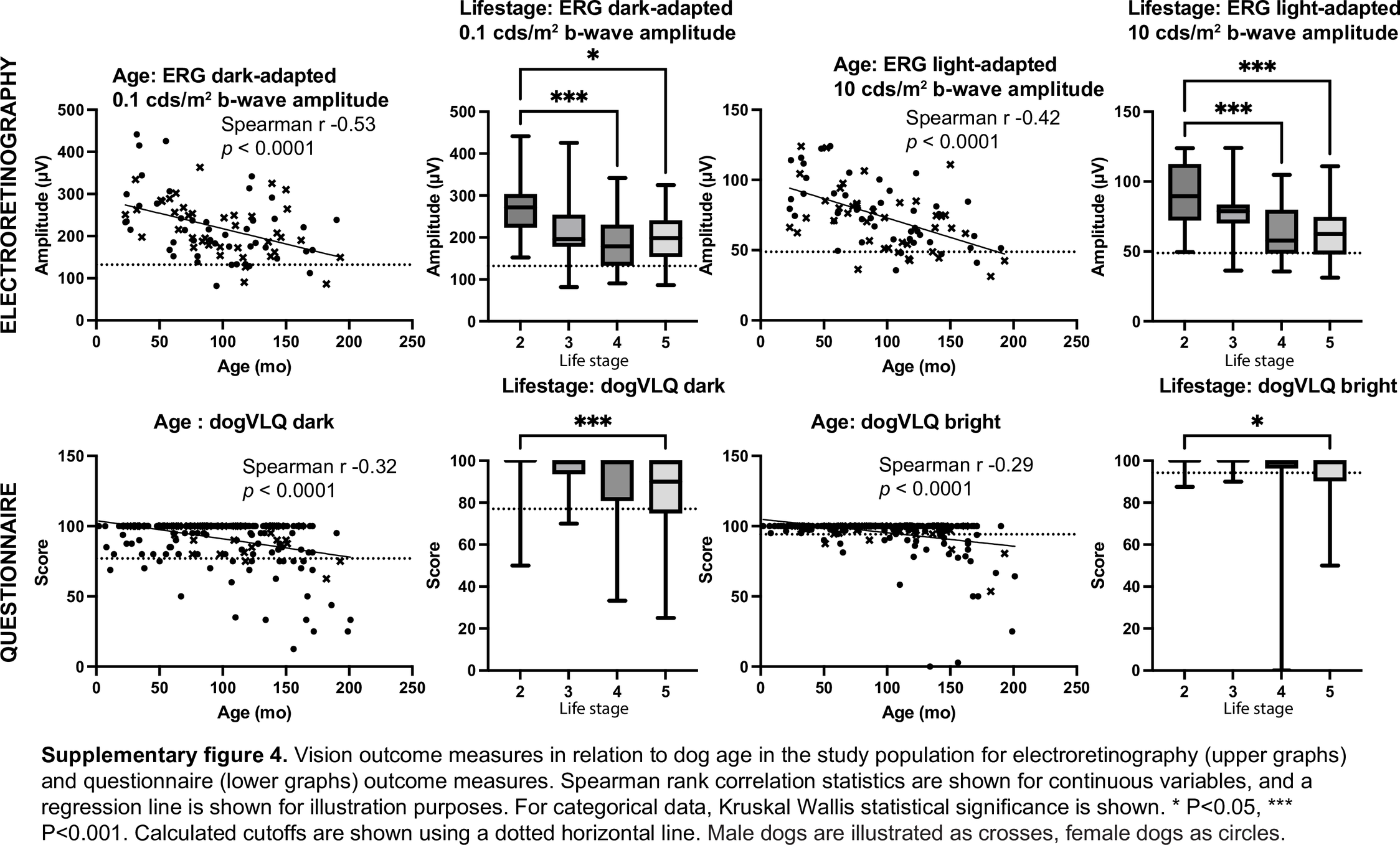

Supplement: S4 Fig — Vision outcome measures in relation to dog age in the study population for electroretinography (upper graphs) and questionnaire (lower graphs) outcome measures. Spearman rank correlation statistics are shown for continuous variables, and a regression line is shown for illustration purposes. For categorical data, Kruskal Wallis statistical significance is shown. * P<0.05, *** P<0.001. Calculated cutoffs are shown using a dotted horizontal line. Male dogs are illustrated as crosses, female dogs as circles. (TIF) [file pone.0310299.s004.tif]
