# Supplementary material for: Dual sensory impairments in companion dogs: Prevalence and relationship to cognitive impairment
Source: PLoS One. 2024 Oct 16;19(10):e0310299. doi: 10.1371/journal.pone.0310299 (PMC11482676; doi:10.1371/journal.pone.0310299)
Supplement: Attachment — Submitted filename: Response to reviewers.docx [file pone.0310299.s006.docx]

PONE-D-24-17815

Dual sensory impairments in companion dogs: prevalence and relationship to cognitive dysfunction.

PLOS ONE

Dear Dr. Mowat,

Thank you for submitting your manuscript to PLOS ONE. After careful consideration, we feel that it has merit but does not fully meet PLOS ONE’s publication criteria as it currently stands. Therefore, we invite you to submit a revised version of the manuscript that addresses the points raised during the review process.

Please submit your revised manuscript by Sep 13 2024 11:59PM. If you will need more time than this to complete your revisions, please reply to this message or contact the journal office at [plosone@plos.org](mailto:plosone@plos.org). When you're ready to submit your revision, log on to [https://www.editorialmanager.com/pone/](https://urldefense.com/v3/__https:/www.editorialmanager.com/pone/__;!!Mak6IKo!O3KhT08LlXms-7JAfbUlhtx3azKY6HaJAWA816yCs9LhWSUNTweeGh-ieoWes3wAc_FZ8y28NqtsCQA$) and select the 'Submissions Needing Revision' folder to locate your manuscript file.

Please include the following items when submitting your revised manuscript:

- A rebuttal letter that responds to each point raised by the academic editor and reviewer(s). You should upload this letter as a separate file labeled 'Response to Reviewers'.
- A marked-up copy of your manuscript that highlights changes made to the original version. You should upload this as a separate file labeled 'Revised Manuscript with Track Changes'.
- An unmarked version of your revised paper without tracked changes. You should upload this as a separate file labeled 'Manuscript'.

If you would like to make changes to your financial disclosure, please include your updated statement in your cover letter. Guidelines for resubmitting your figure files are available below the reviewer comments at the end of this letter.

If applicable, we recommend that you deposit your laboratory protocols in [protocols.io](http://protocols.io) to enhance the reproducibility of your results. [Protocols.io](http://Protocols.io) assigns your protocol its own identifier (DOI) so that it can be cited independently in the future. For instructions see: [https://journals.plos.org/plosone/s/submission-guidelines#loc-laboratory-protocols](https://urldefense.com/v3/__https:/journals.plos.org/plosone/s/submission-guidelines*loc-laboratory-protocols__;Iw!!Mak6IKo!O3KhT08LlXms-7JAfbUlhtx3azKY6HaJAWA816yCs9LhWSUNTweeGh-ieoWes3wAc_FZ8y28OC7a66Q$). Additionally, PLOS ONE offers an option for publishing peer-reviewed Lab Protocol articles, which describe protocols hosted on [protocols.io](http://protocols.io). Read more information on sharing protocols at [https://plos.org/protocols?utm_medium=editorial-email&utm_source=authorletters&utm_campaign=protocols](https://urldefense.com/v3/__https:/plos.org/protocols?utm_medium=editorial-email&utm_source=authorletters&utm_campaign=protocols__;!!Mak6IKo!O3KhT08LlXms-7JAfbUlhtx3azKY6HaJAWA816yCs9LhWSUNTweeGh-ieoWes3wAc_FZ8y28BwJwtuA$).

We look forward to receiving your revised manuscript.

Kind regards,

Maria Santacà
Academic Editor
PLOS ONE

Journal Requirements:

When submitting your revision, we need you to address these additional requirements.

1. Please ensure that your manuscript meets PLOS ONE's style requirements, including those for file naming. The PLOS ONE style templates can be found at
   [https://journals.plos.org/plosone/s/file?id=wjVg/PLOSOne_formatting_sample_main_body.pdf](https://urldefense.com/v3/__https:/journals.plos.org/plosone/s/file?id=wjVg*PLOSOne_formatting_sample_main_body.pdf__;Lw!!Mak6IKo!O3KhT08LlXms-7JAfbUlhtx3azKY6HaJAWA816yCs9LhWSUNTweeGh-ieoWes3wAc_FZ8y285_Mh92Q$) and 
   [https://journals.plos.org/plosone/s/file?id=ba62/PLOSOne_formatting_sample_title_authors_affiliations.pdf](https://urldefense.com/v3/__https:/journals.plos.org/plosone/s/file?id=ba62*PLOSOne_formatting_sample_title_authors_affiliations.pdf__;Lw!!Mak6IKo!O3KhT08LlXms-7JAfbUlhtx3azKY6HaJAWA816yCs9LhWSUNTweeGh-ieoWes3wAc_FZ8y28nxdbdEE$)

*We have reviewed the guidelines and manuscript and figures have been edited per guidelines.*

2. We note that the grant information you provided in the ‘Funding Information’ and ‘Financial Disclosure’ sections do not match.

When you resubmit, please ensure that you provide the correct grant numbers for the awards you received for your study in the ‘Funding Information’ section.

*We have checked this carefully and all listed funding in the “funding information” is correct.*

3. Thank you for stating the following financial disclosure: 
"This work was funded by the National Institutes of Health (K08EY028628, R01AG082907, P30EY016665), the Morris Animal Foundation Mark L. Morris Jr. Investigator Award (D23CA-510), donor funds to the UW-Madison Department of Ophthalmology and Visual Sciences, an unrestricted grant from Research to Prevent Blindness, Inc. to the UW-Madison Department of Ophthalmology and Visual Sciences, and the UW-Madison Women in Science and Engineering and Leadership Institute Vilas Life Cycle Professorship. "

Please state what role the funders took in the study.  If the funders had no role, please state: "The funders had no role in study design, data collection and analysis, decision to publish, or preparation of the manuscript." 
If this statement is not correct you must amend it as needed. 
Please include this amended Role of Funder statement in your cover letter; we will change the online submission form on your behalf.

*We have added this to the cover letter. Thank you.*

[Note: HTML markup is below. Please do not edit.]

Reviewers' comments:

Reviewer's Responses to Questions

**Comments to the Author**

1. Is the manuscript technically sound, and do the data support the conclusions?

The manuscript must describe a technically sound piece of scientific research with data that supports the conclusions. Experiments must have been conducted rigorously, with appropriate controls, replication, and sample sizes. The conclusions must be drawn appropriately based on the data presented.

Reviewer #1: Yes

Reviewer #2: Yes

2. Has the statistical analysis been performed appropriately and rigorously?

Reviewer #1: Yes

Reviewer #2: Yes

3. Have the authors made all data underlying the findings in their manuscript fully available?

The [PLOS Data policy](https://urldefense.com/v3/__http:/www.plosone.org/static/policies.action*sharing__;Iw!!Mak6IKo!O3KhT08LlXms-7JAfbUlhtx3azKY6HaJAWA816yCs9LhWSUNTweeGh-ieoWes3wAc_FZ8y28Q2ee0HE$) requires authors to make all data underlying the findings described in their manuscript fully available without restriction, with rare exception (please refer to the Data Availability Statement in the manuscript PDF file). The data should be provided as part of the manuscript or its supporting information, or deposited to a public repository. For example, in addition to summary statistics, the data points behind means, medians and variance measures should be available. If there are restrictions on publicly sharing data—e.g. participant privacy or use of data from a third party—those must be specified.

Reviewer #1: Yes

Reviewer #2: Yes

4. Is the manuscript presented in an intelligible fashion and written in standard English?

PLOS ONE does not copyedit accepted manuscripts, so the language in submitted articles must be clear, correct, and unambiguous. Any typographical or grammatical errors should be corrected at revision, so please note any specific errors here.

Reviewer #1: Yes

Reviewer #2: Yes

5. Review Comments to the Author

Please use the space provided to explain your answers to the questions above. You may also include additional comments for the author, including concerns about dual publication, research ethics, or publication ethics. (Please upload your review as an attachment if it exceeds 20,000 characters)

Editorial comment:

I have carefully read the manuscript which I find very interesting. I agree with the comments of the two reviewers that I think would improve the manuscript for readers. If you can attend to their points (expecially the problem with the validation of the questionnaires) I am confident I will be able to accept the manuscript.

*We thank the editor for their additional review and are encouraged that overall, the manuscript was reviewed favorably. We hope that the edits made in line with reviewer comments will be acceptable.*

Reviewer #1: The study assesses sensory and cognitive deficits in dogs using both physiological measurements (brainstem auditory evoked potential - BAEP and electroretinography - ERG) and questionnaire assessments. The authors implied at the very beginning that they would explain (make it understood) the relationship between sensory and cognitive function (l20-22: "Dog owners often describe impairments in multiple sensory functions, yet the relationships between sensory and cognitive function in older dogs are not well understood.") However, this explanation did not occur. What the reader received were correlations between impairments that, I think, are already well-known, even among dog owners, as mentioned in the abstract.

My main expectation was that the extensive objective clinical data (which is truly great and important) would validate the subjective assessments of sensory deficits and cognitive capabilities. A questionnaire that reliably measures impairments would be very useful for veterinarians, researchers, and owners alike. However, this question was not the focus of the present analyses.

The highlighted finding is the significant association between dual sensory impairments (hearing and vision) and increased prevalence of cognitive impairments in older dogs, but these findings, are neither novel nor particularly surprising and there is no need for physiological measurements to achieve these results.

*We are disappointed that the reviewer believes that the relationship between sensory and cognitive outcome measures was not well outlined in the manuscript, and that a main outcome should rather be validation of the reported questionnaires using clinical outcomes. Whilst that was a secondary aim (acknowledging that most of the questionnaires are well validated, but to verify that the clinical measures in a population of healthy dogs, in combination with each other, supported the combined questionnaire outcomes, addressed in Supplementary Figures and in Figure 1), our primary aims were:*

1. *To describe the prevalence of sensory impairments (hearing, vision, or both) and cognitive impairment in dogs of a range of ages, cross sectionally. This aim is addressed in Tables 2-5 (describing what proportion of different demographics of dogs had degrees of impairments). We are not aware of literature that previously describes this to-date.*
2. *To determine the prevalence and risk of cognitive impairment in dogs with varying degrees of sensory impairment. This aim is addressed in Tables 6 and 7 (prevalence of cognitive impairment in dogs of different sexes, ages and body sizes with varying degrees of sensory impairments), and in Table 8 (odds ratio of cognitive impairment in dogs aged over 8 years with different degrees of sensory impairments). We are not aware of literature that previously describes this to-date, although we acknowledge that single sensory impairments are associated with cognitive impairment in the literature, and these manuscripts have been cited.*

*To address this reviewer’s concerns, we now include a greater degree of description of prior published validation of the questionnaire outcomes in dogs, and additional justification for validation of the HFQ in healthy dogs cross-sectionally with age compared with BAEP (shown in Figure 1E). See below for additional details.*

*We politely disagree with the statement that “the association between dual sensory impairments (hearing and vision) and increased prevalence of cognitive impairments in older dogs neither novel nor particularly surprising and there is no need for physiological measurements to achieve these results” – we have yet to find literature to support these findings elsewhere, and while it may be intuitive (not “particularly surprising”) – this anecdotal observation does not justify not reporting the outcomes in a rigorous study such as ours. Our goal is to draw as many parallels as possible between companion dogs and humans and justify the study of sensory function in the cognitive domain. There are increasing numbers of cognitive function studies in aging dogs reported (a recent one is from the Dog Aging Project:* [*https://link.springer.com/article/10.1007/s11357-024-01278-x*](https://link.springer.com/article/10.1007/s11357-024-01278-x)*). These studies also aim to draw parallels with aging and cognitive impairment in humans, yet often underplay or ignore the component of sensory function (the entry point for the brain to receive information upon which to act in a cognitive manner). Our studies aim to bring awareness and balance to this nuanced topic, to further augment the companion dog as a human-relevant model for neurologic aging.*

With some difficulties, I could find the correlations between subjective and objective measures, but they were rather weak, as in the case of hearing (Spearman r = -0.27, p = 0.02), or nonexistent, as in the case of vision (at least, "data is not shown" - l292). In the latter case, there was a discrepancy between the subjective and objective measures of sensory impairments, particularly in smaller dogs, where questionnaire results indicated a higher prevalence of sensory impairments compared to clinical evaluations. Unfortunately, this raises questions about the reliability and validity of the applied questionnaires. Although it was mentioned in the text that these questionnaires are validated, perhaps this validation process was not adequate. This issue (i.e. what is the problem with the questionnaires and how could they be developed to improve validity) should be discussed in more detail in the manuscript.

I suggest adding paragraphs in the Methods and Results sections that explicitly investigate the relationship between the questionnaire data and the physiological measures.

*Because the hearing function questionnaire was only previously validated in dogs with otitis, we performed additional validation of it in our healthy aging dog population (presenting data in the manuscript). We have added justification to the results section for the comparison (in healthy dogs) between the HFQ and objective hearing outcome measures:
“Because the original report of the HFQ was validated in dogs clinically affected by otitis [reference Mason et al], we performed association analysis between the HFQ and BAEP threshold (Fig 1E).”*

*We acknowledge that the correlation between BAEP and questionnaire was weak but significant. To further validate the questionnaire as a way to determine hearing impairment cutoffs, however,* *we also (in the same section as the reviewer cited), present sensitivity and specificity analysis for the HFQ. We show that the HFQ is relatively insensitive but quite specific for hearing impairment as defined by the clinical BAEP hearing threshold, placing validity on our “impaired” analyses in subsequent section (they likely represent an underestimate versus an overestimate of impairment prevalence). Sensitivity improves for the HFQ when the impairment cutoff is altered to reflect more substantial hearing loss (>40dB nHL which equates to meaningful hearing loss in humans).*

*With regard to the visual function questionnaires, the cited manuscript provides extensive validation (shown comprehensively in Table 2 in Rogers et. al, Documenta Ophthalmologica 2023, reference 16 in the manuscript), which we did not think was relevant to recapitulate in this manuscript. We do, however, go on after this citation to describe the sensitivity and specificity of the bright and dim light dogVLQ in detail, supporting validity for our impairment cutoffs, which was a primary goal of this analysis. Similar to the HFQ, the vision questionnaires had low sensitivity but high specificity for impairment compared with clinical measures.*

*We fully acknowledge that a dog owner questionnaire is not going to represent the optimal method with which to study (by proxy) a clinical phenomenon. The convenience factor and the statistical power this can garner is important, however and highly applicable to large-scale epidemiologic studies. We have augmented an existing limitation in the discussion section to read (new text underlined):
“However, all our utilized questionnaires have been compared to clinical outcome measures and while they are relatively insensitive to the extent of impairment we determined using clinical measures, they have suitable validity to detect meaningful impairment”*


The methodological section lacks adequate detail, particularly with regard to the cognitive dysfunction and sensory impairments questionnaires. Only references are mentioned.

*The references cited are manuscripts that in describe in detail the development and validation of these questionnaires. More detail is provided in the specific reviewer comment below.*

Discussion: As promised in the abstract, hypotheses on the biological background ("understanding") of the possible reciprocal relationship between sensory and cognitive deficits would improve the text.

*We had a small section of discussion dedicated to the reciprocal relationship between sensory and cognitive impairments, as detailed below. We have augmented this section slightly to reflect a greater discussion of the relationships between sensory and cognitive dysfunctions (new text underlined).*

*“No studies to-date have examined the relationship between cognition and combined impairments of multiple senses in dogs. There is strong evidence of such a relationship in humans. Compared with no sensory impairment or sole sensory impairment, people with dual or multiple sensory impairments are predisposed to a greater degree of cognitive decline [28, 63, 64], and more extensive cortical amyloid beta deposition [65], a critical pathologic hallmark of Alzheimer’s disease and related dementias. However, the relationship between sensory and cognitive impairments is not definitively unidirectional – for example, a bidirectional relationship between impairments in vision and cognition has been suggested by metanalysis of human studies [21]. Patients with dementia are at increased risk of developing vision[21] and hearing impairments [22]. Conversely, there is a higher risk of dementia in patients with sensory morbidities including vision impairment[23-28], hearing impairment[28-31], and olfactory impairment[32, 33]. Because the relationship between dementia and sensory impairment is bidirectional, this supports the “common cause” hypothesis which suggests that shared pathways of external factors influence the incidence of multiple disorders [66]. The study of these external factors may be most efficiently studied using a relevant animal model, such as dogs.”*

Other comments:
- l20 (Canis...) - The Latin name should begin with a capital C.
*We have amended this -thank you for picking that up!*

- l49 and elsewhere: "cognitive impairment" is used as a synonym for "cognitive dysfunction" in the text. I suggest consistently using the same terminology throughout the manuscript to ensure clarity.
*We have revised the manuscript to use the word “impairment” throughout. There is heterogeneity in the cited literature, our apologies for any additional confusion.*

- l84 Study participants: Add the number of participants in this section.
*Our apologies for the omission. We have now described how many questionnaires were distributed to participants. Actual participating n-numbers continue to be described in the results section.*

- l98-100: Provide more information about the questionnaires (e.g., number of questions, example questions, how these tools were validated), so readers do not need to look them up in the references. This will also help clarify whether the present study could improve the validation, especially if there were gaps in previous literature. If gaps are identified, mention them in the Introduction.
*These questionnaires are well validated, and more information has been provided to support this in the methods. New text is underlined below:
“Information collected included demographics (date of birth or approximate age if date unknown, breed if purebred, sex, neutering status, body weight category), and standardized questionnaire responses regarding dog hearing (the 8-question dog hearing function questionnaire; HFQ [references]), vision (the 17-question dog variable lighting questionnaire; dogVLQ [references]), and cognitive impairment (the 17-question CAnine DEmentia Scale; CADES [references]). These questionnaires have been extensively validated against objective measures in prior studies. In the original report, the HFQ was validated in dogs with otitis externa or otitis media, using brainstem auditory evoked potential threshold testing (similar to our methodology described below) [references]. The recently reported dogVLQ was extensively validated in healthy dogs against the gold-standard, objective measure of retinal function, electroretinography [references]. The CADES is extensively validated against other canine cognitive questionnaires [references], clinical cognitive tests [references], and neurodegeneration biomarkers [references].”

The knowledge gaps that we aimed to address are outlined in the introduction (new text is underlined):
“ …although general population prevalence of these sensory impairments has not been reported”
“To-date, analysis of cognitive and sensory relationships in companion dogs are limited to a single sense”

We also justify regression of the HFQ vs. the BAEP threshold in Fig 1E in the text, as the prior research reporting this question did so in unhealthy dogs:
“Because the original report of the HFQ was validated in dogs clinically affected by otitis[reference], we performed association analysis between the HFQ and BAEP threshold (Fig 1E), to validate it further in dogs with healthy ears.”*

- l150: Include the weight category of large and small dogs, so readers do not need to look it up in the reference.
*We have modified this sentence to clarify (new text underlined): “To account for potential variance in lifespan between smaller (<50kg) and large (>50kg) dogs [reference],”*

- l160: "largest group of young dogs" - Clarify that "large" refers to the sample size and not the body size, if this is the case.
*In light of this comment we have changed this to “most numerous” to be explicitly clear*

- l169-170: "using standard methods" - Briefly describe what these methods are.
*We have augmented this section as recommended (new text underlined): “Briefly, we calculated true and false positive and negative rates of impairment based on the function questionnaires compared with the gold standard clinical tests; sensitivity was calculated as the number of true positives divided by the sum of the true positives and the false negatives; specificity was calculated as the number of true negatives divided by the sum of the true negatives and the false positives. We also calculated the Youden index of the questionnaires using standard methods (sensitivity plus specificity, minus 100) [reference].”*

- l185: Explain what can be learned from the list of diseases. How representative are these dogs? What kind of population do they represent?
*We acknowledge that many dogs had identifiable mild neurologic findings on clinical examination. This is discussed in the discussion section limitations:
“Many dogs in the study had neurologic abnormalities identified on clinical examination, which may affect owners’ interpretation of hearing, vision and cognitive impairment in the home environment. However, these abnormalities were overall mild and potentially transient. The prevalence of neurologic abnormalities in our group of dogs was in line with prevalence in a similar study in apparently healthy dogs [reference].”*

- l483: Clarify if there was financial support for this study.
*This is not permitted to be listed in the manuscript body per journal guidelines. Financial disclosures and funding are provided during the submission process and are presumably aligned with the manuscript once it is formatted for publishing*

Reviewer #2: In this manuscript the authors assessed dog vision and hearing clinically and via questionnaires, to assess the relationships between vision and/or hearing impairments and cognitive impairments. Their results indicate a potential earlier onset of vision impairment compared with hearing impairment.

The authors also established sensory impairment cutoffs based on 161 young dogs then used this to evaluate sensitivity and specificity of the questionnaire. Their goal is to provide a methodology to detect impairment using objective clinical tests using standard methods.

The research question is timely and the approach of the study is also valid. I applaud the authors for [not] administering any sedation or anxiolytics to get better quality data and that they assigned the dogs by life stages.

The presentation of the results is very informative and detailed.

I only have a few minor comments:

line 107 A subset of dogs (n = 91) participated. --It is not clear here in the text what does this refer to, subset of what?
*Our apologies for any confusion – we have amended this sentence to clarify that the subset was of those that had questionnaire data obtained:* *A subset of dogs that had owner questionnaire data obtained”*

line 146 dak adaptation –typo
*Corrected – thank you*

The sample size is too small to evaluate the effect of life stages, body size or cephalic index (and their possible interactions with sufficient resolution), but the study is excellent for validating the questionnaires.

*We thank the reviewer for their positive comments*

What is the difference between table 2 and 3? They seem to be same data with minor differences ( one with n=75 the other with n=76)
*Our apologies that this was not more obvious – they show light-adapted responses (Table 2) and dark-adapted responses (table 3) separately, as we did not want to amalgamate these results. We have clarified this in the title/legend of the table.*

I have the same question for table 4 and 5. It is not clear what is the difference between them based on the legend,, apart from sample size (238 vs 227)
*Our apologies that this was not more obvious – they show bright light visual responses (Table 4) and dim light visual responses (Table 5) separately, as we did not want to amalgamate these results. We have clarified this in the title/legend of the table.*

6. PLOS authors have the option to publish the peer review history of their article ([what does this mean?](https://urldefense.com/v3/__https:/journals.plos.org/plosone/s/editorial-and-peer-review-process*loc-peer-review-history__;Iw!!Mak6IKo!O3KhT08LlXms-7JAfbUlhtx3azKY6HaJAWA816yCs9LhWSUNTweeGh-ieoWes3wAc_FZ8y28OjL5J0o$)). If published, this will include your full peer review and any attached files.


If you choose “no”, your identity will remain anonymous but your review may still be made public.


**Do you want your identity to be public for this peer review?** For information about this choice, including consent withdrawal, please see our [Privacy Policy](https://urldefense.com/v3/__https:/www.plos.org/privacy-policy__;!!Mak6IKo!O3KhT08LlXms-7JAfbUlhtx3azKY6HaJAWA816yCs9LhWSUNTweeGh-ieoWes3wAc_FZ8y28--B2Pi8$).

Reviewer #1: No

Reviewer #2: **Yes:** Dora Szabo, PhD

[NOTE: If reviewer comments were submitted as an attachment file, they will be attached to this email and accessible via the submission site. Please log into your account, locate the manuscript record, and check for the action link "View Attachments". If this link does not appear, there are no attachment files.]

While revising your submission, please upload your figure files to the Preflight Analysis and Conversion Engine (PACE) digital diagnostic tool, [https://pacev2.apexcovantage.com/](https://urldefense.com/v3/__https:/pacev2.apexcovantage.com/__;!!Mak6IKo!O3KhT08LlXms-7JAfbUlhtx3azKY6HaJAWA816yCs9LhWSUNTweeGh-ieoWes3wAc_FZ8y283t4NQko$). PACE helps ensure that figures meet PLOS requirements. To use PACE, you must first register as a user. Registration is free. Then, login and navigate to the UPLOAD tab, where you will find detailed instructions on how to use the tool. If you encounter any issues or have any questions when using PACE, please email PLOS at [figures@plos.org](mailto:figures@plos.org). Please note that Supporting Information files do not need this step.

*In compliance with data protection regulations, you may request that we remove your personal registration details at any time.*  [*(Remove my information/details)*](https://urldefense.com/v3/__https:/www.editorialmanager.com/pone/login.asp?a=r__;!!Mak6IKo!O3KhT08LlXms-7JAfbUlhtx3azKY6HaJAWA816yCs9LhWSUNTweeGh-ieoWes3wAc_FZ8y28SgGSK8s$)*. Please contact the publication office if you have any questions.*
